# The CDK9 Tail Determines the Reaction Pathway of Positive Transcription Elongation Factor b

**DOI:** 10.1016/j.str.2012.08.011

**Published:** 2012-10-10

**Authors:** Sonja Baumli, Alison J. Hole, Lan-Zhen Wang, Martin E.M. Noble, Jane A. Endicott

**Affiliations:** 1 Northern Institute for Cancer Research, Newcastle University, Newcastle upon Tyne NE2 4HH, UK

## Abstract

CDK9, the kinase of positive transcription elongation factor b (P-TEFb), stimulates transcription elongation by phosphorylating RNA polymerase II and transcription elongation factors. Using kinetic analysis of a human P-TEFb complex consisting of CDK9 and cyclin T, we show that the CDK9 C-terminal tail sequence is important for the catalytic mechanism and imposes an ordered binding of substrates and release of products. Crystallographic analysis of a CDK9/cyclin T complex in which the C-terminal tail partially blocks the ATP binding site reveals a possible reaction intermediate. Biochemical characterization of CDK9 mutants supports a model in which the CDK9 tail cycles through different conformational states. We propose that this mechanism is critical for the pattern of CTD Ser2 phosphorylation on actively transcribed genes.

## Graphical abstract

**Graphical Abstract:**

**Figure:**
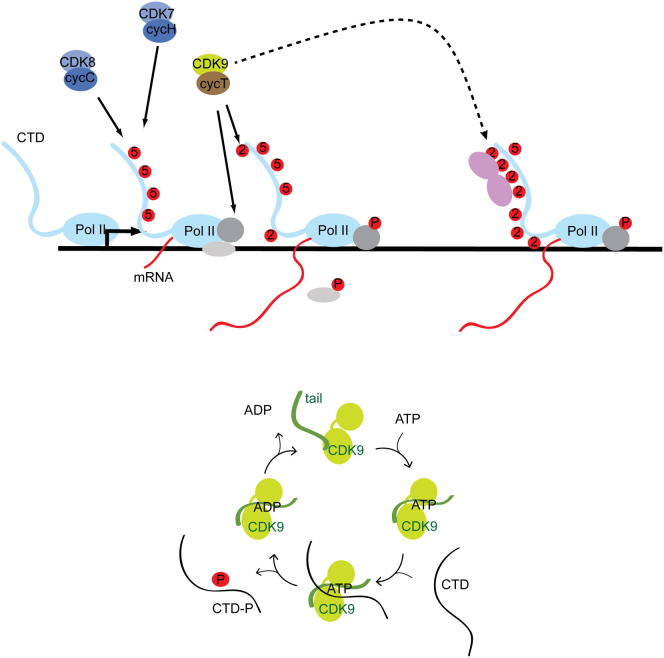

**Highlights:**

► The CDK9 C-terminal tail is important for the catalytic mechanism ► CDK9 phosphorylates the CTD in a nonprocessive manner ► The CDK9 tail folds over the ATP binding site during the catalytic cycle

## Teaser

CDK9 kinase stimulates transcription elongation and is a drug target for cancer therapy. Baumli et al. describe the conformational states of CDK9 as it places multiple phosphorylations on substrates. The observed mechanism is critical for the phosphorylation pattern that is generated on actively transcribed genes.

## Introduction

Transcription of protein coding genes by RNA polymerase II (Pol II) is a highly regulated process. Transcription is regulated by the recruitment of Pol II not only during transcription initiation but also during elongation. On many genes, Pol II pauses during early transcription elongation. Genome-wide studies in murine, human, and *Drosophila* cells have revealed that such promoter proximal pausing is a widespread mechanism that regulates the rate of gene transcription (Core et al., 2008; Nechaev and Adelman, 2011; Price, 2008).

Promoter proximal pausing is reversed by the activity of P-TEFb, a complex of cyclin-dependent kinase 9 (CDK9) and cyclin T1 or T2. The enzyme phosphorylates the elongation factors DSIF (5,6-dichlorobenzimidazole 1-β-*D*-ribofuranoside [DRB]-sensitivity-inducing factor), and NELF (negative elongation factor) (Peterlin and Price, 2006; Zhou and Yik, 2006) as well as Ser2 within the heptad repeat sequence in the C-terminal domain (CTD) of the largest Pol II subunit. These phosphorylation events release paused Pol II and allow productive transcription elongation. The CTD is a binding platform for the recruitment of transcription, RNA processing, and histone-modifying factors. CDK7–CDK9, CDK12, and CDK13 phosphorylate specific residues within the CTD heptad repeats. Changes in the phosphorylation pattern are linked to progression through the different stages of transcription and serve to recruit the appropriate transcription factors (Buratowski, 2009; Meinhart et al., 2005). Although it is clear that different CTD residues are phosphorylated at distinct stages in the transcription cycle, it remains to be determined how many of the 52 repeats are phosphorylated and whether patterns of phosphorylation occur.

The structures of the conserved core domains of cyclins T1 and T2 and of a CDK9/cyclin T1 complex provide insights into P-TEFb organization (Anand et al., 2007; Baumli et al., 2008). CDK9 adopts a typical protein kinase fold, whereas cyclin T has a canonical cyclin structure. The CDK9-cyclin T1 interface is restricted to the N-terminal kinase lobe and is notably smaller than in other CDK/cyclin complexes, leading to greater conformational flexibility of the kinase structure. Complexes between CDK9/cyclin T and HIV-1 TAT or ATP-competitive inhibitors reveal that CDK9 can undergo structural changes in response to regulator binding (Baumli et al., 2008, 2010; Bettayeb et al., 2010; Tahirov et al., 2010). Such conformational plasticity has been documented for a number of protein kinases and is a characteristic of the protein kinase fold that is often exploited by regulatory factors (Endicott et al., 2012; Huse and Kuriyan, 2002).

**Figure 1:**
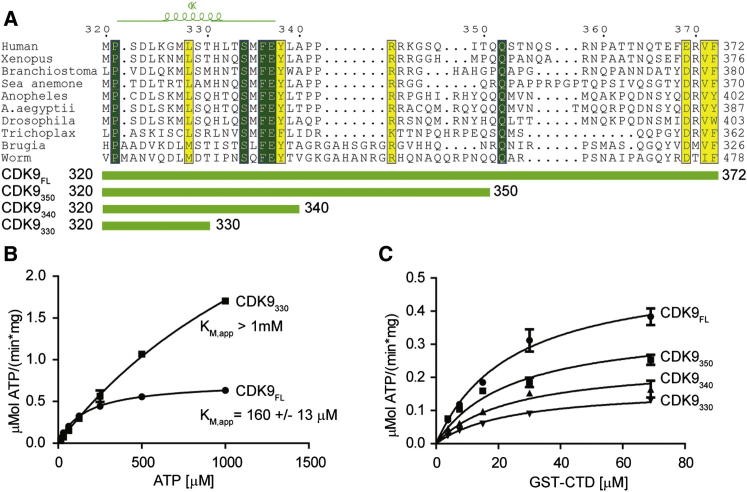
The C Terminus of CDK9 Influences Kinase Activity (A) Sequence alignment of the C-terminal region of CDK9. Identical residues are highlighted in green, and chemically equivalent residues are in yellow. The location of the most C-terminal CDK9 α helix is shown above the alignment. The constructs used in (B) and (C) are identified below the alignment. (B) Activity of CDK9_FL_/cyclin T and CDK9_330_/cyclin T assayed in the presence of increasing amounts of ATP. (C) Activity of various CDK9/cyclin T complexes with increasing concentrations of GST-CTD. CDK9 variants are labeled as shown in (A). All measurements were done in triplicate and reproduced in independent experiments. Error bars indicate standard errors (SEs). For a summary of the apparent kinetic parameters, see Table S1. See also Figure S1.

In addition to the protein kinase fold, CDK9 has a C-terminal tail (residues 331–372; Figure 1A) with a largely nonconserved sequence. This tail is required for the nuclear import of P-TEFb (Napolitano et al., 2003) and for high-affinity binding to HIV TAT/TAR (Garber et al., 2000). CDK9/cyclin T nuclear import and association of CDK9/cyclin T/TAT with TAR RNA are dependent on autophosphorylation of several sites within the tail (Garber et al., 2000). However, the phosphorylation status of these sites does not affect CDK9 activity toward the Pol II CTD measured in vitro (Baumli et al., 2008).

To understand how CDK9 phosphorylates the multiple repeats in the Pol II CTD, we focus here on the kinetic mechanism. We show that the CDK9 C-terminal tail defines the catalytic mechanism. It ensures that CDK9-catalyzed phosphotransfer proceeds through sequentially ordered formation of a ternary enzyme/ATP/CTD complex. We present the structure of P-TEFb, including the CDK9 C-terminal tail, which was truncated in previous structures. We find that binding of the ATP-competitive inhibitor DRB selectively stabilizes a CDK9 conformation in which the C-terminal tail is structured and folds over the ATP binding site. Analysis of CDK9 C-terminal tail mutants show that this bound conformation is a prerequisite for the observed ordered kinetic mechanism and represents a possible reaction intermediate. A consequence of this mechanism is that the phosphorylated CTD is released from the CDK9 active site after each catalytic turnover. We propose that this mechanism affects the distribution of Ser-2 phosphorylated sites on the Pol II CTD and thereby specifies recruitment of CTD-binding proteins.

## Results

### The CDK9 C-Terminal Tail Is Important for CDK9 Kinase Activity

To analyze the function of the CDK9 C-terminal sequence, we expressed and purified full-length human CDK9 (residues 1–372, CDK9_FL_) and compared its activity with that of the truncated CDK9 construct CDK9_330_ (residues 1–330) for which structural information is available. Both forms of CDK9 were bound to truncated cyclin T (residues 1–288, cyclin T) and kinase activity was measured against a GST-tagged version of the human Pol II CTD (GST-CTD). At an ATP concentration of 100 μM, CDK9_330_ has only 30% of the kinase activity of the full-length protein (Figure S1A available online). Further enzymatic analysis revealed that this difference in activity does not result from differences in affinity toward GST-CTD, because both CDK9_330_ and CDK9_FL_ have very similar K_M,app_ (apparent K_M_ value at a fixed concentration of the second substrate) values for this substrate (21.3 ± 3.7 μM versus 24.3 ± 2.7 μM, respectively; Figure S1B). In contrast, CDK9_FL_ shows a much lower K_M,app_ toward ATP than truncated CDK9_330_ (K_M,app ATP_ CDK9_FL_ 160 ± 13 μM; K_M,app ATP_ CDK9_330_ > 1 mM; Figure 1B; Table S1). The tail-length dependence of only the maximal velocity (V_max_), and not K_M_, with respect to the Pol II CTD indicates that the C-terminal sequence is not involved in recognition of the peptide substrate. Furthermore, the dependence of K_M_ as well as V_max_ with respect to the ATP substrate indicates that the C-terminal tail influences ATP binding and catalysis (Cornish-Bowden, 2004).

To analyze which region of the C-terminal tail is important for the observed change in kinase activity, we constructed a series of CDK9 truncations (Figure 1A). Analysis of these constructs at an ATP concentration of 100 μM revealed a gradual loss of activity toward GST-CTD as the C-terminal tail is shortened (Figure 1C; Table S1). Consistent with the mechanism described above, all truncations show similar K_M,app_ values, but a gradual decrease in V_max,app_ toward the GST-CTD substrate. These results demonstrate that the length of the C-terminal tail affects the apparent kinetic parameters of CDK9.

### Deletion of the CDK9 Tail Changes the Kinetic Mechanism

To better understand the molecular mechanism of phosphorylation by CDK9, we analyzed kinase activity by varying the concentrations of both GST-CTD and ATP substrates. An analysis of the initial rate experiments demonstrates that, in agreement with what is generally observed for protein kinases (Adams, 2001), the reaction curves are consistent with a ternary complex mechanism, in which a complex containing both substrates bound to the kinase is formed during catalysis (Table 1; Figure S2).

**Table 1:** Overall Kinetic Parameters

	CDK9_FL_/cyclin T
K_M, ATP_	329 ± 55 μM
K_M, CTD_	115 ± 17 μM
K_ATP_	21 ± 9 μM
K_cat_/K_M,ATP_	0.55 min^−1^μM^−1^
K_cat_/K_M,CTD_	1.6 min^−1^μM^−1^
K_cat_	178 ± 19 min^−1^

K_ATP_, K_M, CTD_, and K_M, ATP_ correspond to the ATP dissociation constant and Michaelis constants for GST-CTD and ATP, respectively. The corresponding raw data are shown in Figure S2. Akaike's information criterion as implemented in Graphpad Prism (www.graphpad.com) was used to distinguish between ternary complex and substituted enzyme mechanisms. The data are compatible with the ternary complex mechanism (probability: 96%).

To investigate whether the order of substrate binding and release is important for the catalytic mechanism, we carried out product inhibition experiments using low concentrations of ADP. If both substrates bind to the kinase in a random order, then the ADP product should act as a competitive inhibitor with respect to both the ATP and the GST-CTD substrates. If substrate binding to the enzyme is ordered, then ADP should not compete with both substrates, and the inhibition mechanism toward either one should be dependent on the sequence of substrate addition (Cornish-Bowden, 2004).

**Figure 2:**
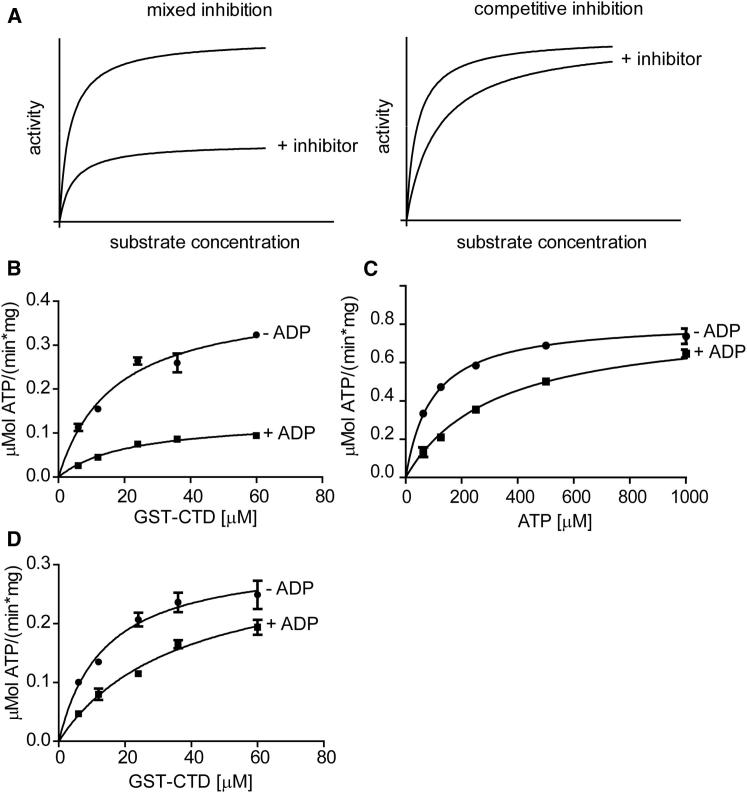
The CDK9 Tail Is Required for the Ordered Substrate Addition Catalytic Mechanism (A) Theoretical model curves for mixed and competitive inhibition assuming the same K_M_, K_i_, and V_max_ in both cases. (B) Activity of CDK9_FL_/cyclin T in the absence and presence of 2.5 μM ADP, in the presence of 100 μM ATP and increasing amounts of CTD. (C) Activity of CDK9_FL_/cyclin T in the absence and presence of 2.5 μM ADP, in the presence of 36 μM CTD and increasing amounts of ATP. (D) Activity of CDK9_330_/cyclin T in the absence and presence of 2.5 μM ADP, in the presence of 100 μM ATP and increasing amounts of CTD. All measurements were done in triplicate and reproduced in independent experiments. Error bars in (B)–(D) represent SEs. See also Figure S3.

These experiments revealed that ADP is a potent CDK9 inhibitor with a low micromolar K_*i*_ (Figure 2B). ADP inhibits CDK9_FL_ with respect to the GST-CTD substrate by decreasing V_max_ and increasing K_M_, which is characteristic of a mixed inhibition mechanism (Figures 2A and 2B). However, ADP acts as a competitive CDK9 inhibitor with respect to the substrate ATP (Figures 2A and 2C). These results are consistent with a reaction that proceeds through an ordered recruitment of substrates, with ATP being the first substrate to be bound and ADP the second product to be released. Of interest, a different behavior is observed for the CDK9 C-terminal deletion: ADP inhibits competitively with respect to both substrates (Figure 2D), indicating that they bind to CDK9_330_ in a random order. Taken together, these results suggest that the CDK9 C-terminal tail ensures that the reaction follows a compulsory order ternary complex mechanism during which ATP binds first to the kinase followed by the CTD and that following catalysis, the phosphorylated CTD is the first product to be released.

### The CDK9 C-Terminal Tail Becomes Structured upon Binding to an Active Kinase Conformation

**Figure 3:**
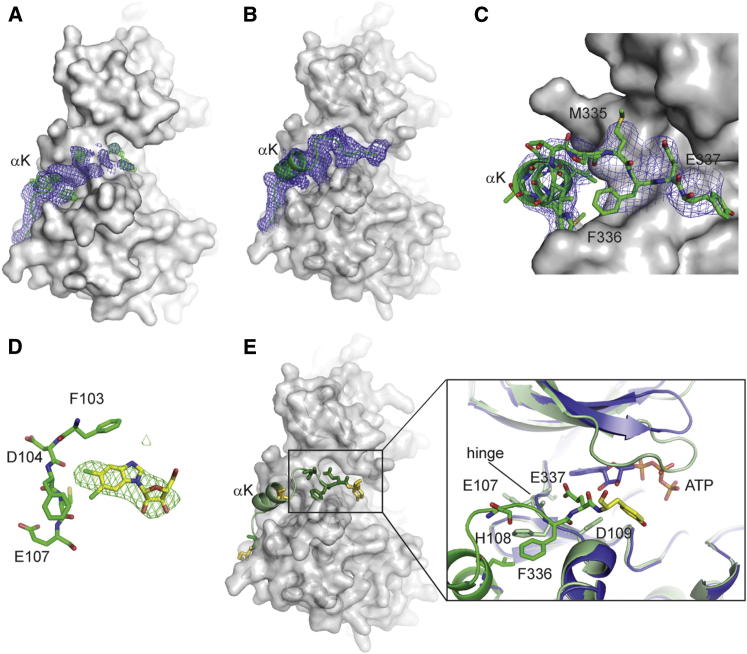
Structure of the CDK9 C-Terminal Tail (A) The final electron density map corresponding to additional C-terminal residues present in CDK9_FL_ bound to cyclin T_259_ in the absence of DRB or AMPPNP is shown as a blue mesh at a contour level of 1σ. The difference density is shown as a green mesh at a contour level of 3σ. The CDK9 core structure is displayed as a solvent-accessible surface representation in gray. (B) Same view as in (A) to show the electron density corresponding to the CDK9 C-terminal sequence present in the structure of CDK9_FL_/cyclin T_259_ bound to DRB. The electron density map is drawn as a blue mesh and contoured at 1σ. (C) Detailed view of the interactions between the CDK9 C-terminal residues and the hinge sequence present in the CDK9_FL_/cyclin T_259_/DRB complex. The solvent-accessible surface of CDK9_330_ is drawn in gray and selected residues are labeled. The electron density map of the refined structure is drawn at a contour level of 1σ. (D) The difference density map corresponding to the DRB binding site in CDK9_FL_ is shown at a contour level of 3σ. Residues of the CDK9_FL_ hinge region (from Phe103 to Glu107) are also shown. (E) Interactions of Phe336 and Glu337 with the CDK9 hinge region. C-terminal residues that are identical across species are drawn as sticks in green, and equivalent residues are in yellow. CDK9/cyclin T_259_ (PDB 3BLH) bound to ATP is superposed in blue for comparison. See also Figure S4.

To date, P-TEFb structures have been determined using truncated CDK9 and cyclin T that were engineered to improve crystal quality. In these structures, electron density for the C-terminal sequence of CDK9 is either missing after residue 325 (Baumli et al., 2008) or extends away from the CDK9 fold and adopts a structure that is determined by crystal contacts (Tahirov et al., 2010; Figure S4). In order to understand the molecular mechanism by which the C-terminal tail controls CDK9 activity, we solved the structure of apo CDK9_FL_/cyclin T_259_ (residues 1–259) at a resolution of 3.2 Å (Table 2; Figure 3A). As expected, the cores of both subunits of the complex closely resemble the previously published CDK9_330_/cyclin T_259_ structure (Baumli et al., 2008). Additional electron density is observed for CDK9 residues 326–327, which form an α-helical turn at the back of CDK9. The electron density gradually weakens after residue 327, and further residues could not be built with confidence. This result indicates that the CDK9 C-terminal tail is inherently flexible.

**Table 2:** Data Collection and Refinement Statistics

	CDK9_FL_/cyclin T/DRB	CDK9_FL_/cyclin T
**Data Collection**		

Space group	H3	H3

**Cell dimensions**		

*a*, *b*, *c* (Å)	175.1, 175.1, 101.3	174.3, 174.3, 97.9
α, β, γ (°)	90, 90, 120	90, 90, 120
Resolution (Å)	60.69–3.6 (3.79–3.6)	46.6–3.2 (3.38–3.2)
*R*_merge_	0.12 (0.77)	0.051 (0.514)
*I* / σ*I*	4.7 (1.4)	11.7 (1.5)
Completeness (%)	98.2 (99.3)	98.9 (98.2)
Redundancy	2.1 (2.1)	2.8 (2.9)

**Refinement**		

Resolution (Å)	49.87–3.6	46.60–3.21
Number of reflections	13,155	17,257
*R*_work_ / *R*_free_	19.2 / 23.0	20.1 / 23.32
Number of protein atoms	4,700	4,534
B-factors (Å^2^)	125.7	144

**rmsd**		

Bond lengths (Å)	0.008	0.008
Bond angles (°)	1.073	1.456

Values in parentheses are for highest-resolution shell.

We recently showed that the CDK9-specific inhibitor DRB is a more potent inhibitor of CDK9_FL_ than of CDK9_330_, and stabilizes CDK9_FL_ (Baumli et al., 2010). By soaking DRB into preformed CDK9_FL_/cyclin T_259_ crystals, we aimed to exploit this stabilizing effect to observe the CDK9 C-terminal tail residues. The resulting crystal structure, solved at 3.6 Å resolution, shows unambiguous electron density for the inhibitor and 11 additional CDK9 C-terminal residues as compared with the structure of CDK9_FL_/cyclin T_259_ (Table 2; Figures 3B–3D). Superposition of the two DRB-bound structures shows that the DRB binding mode is unaffected by the C-terminal tail, and that apart from some CDK9 flexibility at the tip of the glycine-rich loop, the structures close to the ATP-binding site (as seen in PDB: 3BLH) are very similar (root mean-square deviation [rmsd] = 0.946 over all CDK9 atoms when compared with PDB: 3MY1) (Baumli et al., 2010; Figure 3E). CDK9 adopts a closed state upon DRB binding in which the N-terminal lobe is rotated by 8° with respect to the apo or ATP-bound structure.

This closed state is stabilized in the context of CDK9_FL_ by folding of the C-terminal tail over the ATP binding site (Figures 3B and 3D). The CDK9 C-terminal sequence makes no direct contacts with DRB. However, the DRB-bound CDK9 conformation is stabilized by Phe336 and Glu337, two strictly conserved residues that form a clamp around the CDK9 hinge region, close to where ATP is observed in PDB: 3BLH (Figure 3E). Phe336 inserts into a hydrophobic pocket just before the hinge, and Glu337 inserts into the ATP binding site. Correct alignment of the N- and C-terminal lobes is an important mechanism of regulation for many kinases (Huse and Kuriyan, 2002). The orientation of the CDK9 N- and C-terminal lobes induced by DRB binding is characteristic of protein kinase and protein kinase/substrate complexes that are poised for catalysis. This structure may be further stabilized by the binding of the C-terminal tail. These observations are consistent with a role for the CDK9 C-terminal tail in the catalytic mechanism.

### Conformational Cycling of the CDK9 C-Terminal Tail Is Required for the CDK9 Catalytic Cycle

**Figure 4:**
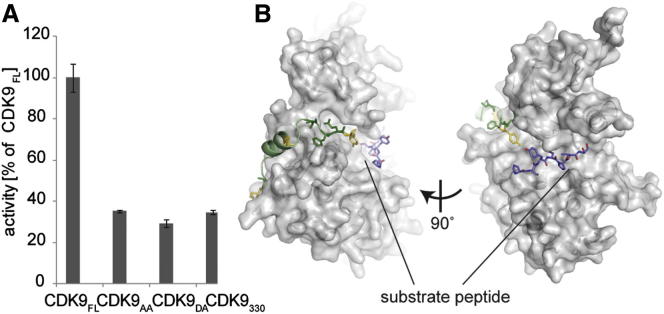
Flexibility of the CDK9 C Terminus Is Important for Kinase Activity (A) Activity of CDK9_FL_/cyclin T toward GST-CTD compared with complexes containing the CDK9 clamp mutants CDK9_AA_ and CDK9_DA_, and the CDK9 truncation CDK9_330_. All measurements were done at 100 μM ATP and 24 μM GST-CTD in triplicate and reproduced in independent experiments. Error bars represent SEs. (B) Model showing that binding of a substrate peptide (lilac) is compatible with the bound conformation of the CDK9 C-terminal region. In this model, Ser2 of the heptad repeat occupies the phosphotransfer position. The cartoon-and-stick model is as described in Figure 3E.

To test whether the bound conformation of the C-terminal tail that we observed in our structural studies is relevant for the kinase mechanism, we mutated the clamp-forming residues Phe336 and Glu337. In one mutant, each residue was substituted by alanine (CDK9_AA_), and in another mutant, Phe336 and Glu337 were changed to an aspartate and alanine, respectively (CDK9_DA_). Mutation to an aspartate introduces a charged residue that can no longer be accommodated in the hydrophobic pocket. At low ATP concentrations, both mutants exhibited only ∼30% of the kinase activity of the full-length protein toward GST-CTD, demonstrating that these two conserved residues are required for optimal CDK9 activity. The low activity observed for the clamp mutants under these conditions is similar to that of CDK9_330_ (Figure 4A). As observed with CDK9_330_, ADP acts as a competitive inhibitor of CDK9_DA_ with respect to the CTD substrate (Figure S3). This result indicates that mutation of the clamp residues changes the CDK9 kinetic pathway despite the presence of the C-terminal tail. Taken together, our results suggest that the bound state of the C-terminal tail and the associated kinase conformation observed in the CDK9_FL_/cyclin T_259_/DRB complex represents a conformation that is adopted during the reaction cycle.

To confirm that the position of the C-terminal tail in its bound conformation does not interfere with binding of either substrate and thus is compatible with substrate turnover, we modeled ATP and a substrate peptide into this structure. Figures 3E and 4B show that the conformation of the C-terminal tail is compatible with binding of both ATP and the substrate peptide, which binds against the activation segment at the other end of the ATP binding pocket (Baumli et al., 2008).

The only close contact occurs between ATP and the glycine-rich loop (CDK9 residues 26–33), a mobile region that has been shown to adopt a number of different conformations (Baumli et al., 2008, 2010; Tahirov et al., 2010). In this model, the ATP binding site is fully enclosed by the substrate peptide from one side and the C-terminal tail from the other side. The model also suggests that the C-terminal sequence has to unlock from this position to allow the exchange of ADP and ATP. This analysis suggests that a degree of conformational flexibility of the C-terminal tail, as observed in the apo and the ATP-bound crystal structures, is required for CDK9 activity.

## Discussion

**Figure 5:**
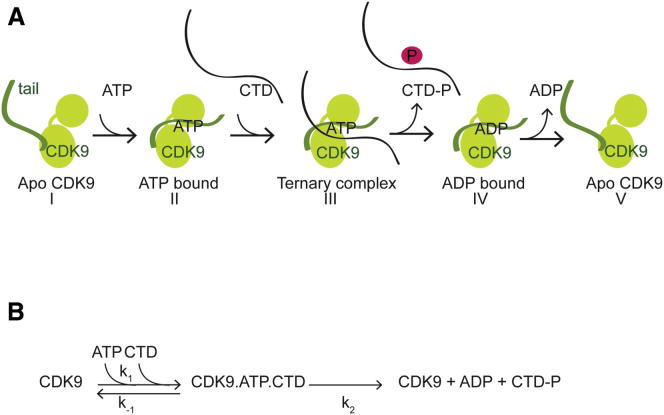
Model of the CDK9 Catalytic Cycle (A) Model of the CDK9 conformational states adopted during the catalytic cycle. The cyclin subunit is omitted from the scheme for clarity. (I) Apo CDK9 has a flexible C-terminal tail. ATP is the first substrate bound to CDK9. (II) Upon ATP binding, the CDK9 C-terminal tail adopts an ordered structure, partially covering the ATP binding site. (III) The Pol II CTD binds to the CDK9/ATP complex to form a ternary complex that is competent for phosphotransfer. (IV) After phosphorylation, the CTD substrate is released. (V) ADP is released and the kinase returns to its apo state with a flexible C-terminal tail. (B) Reaction scheme. k_1_ and k_-1_ refer to the rate constants for ternary complex formation, and k_2_ is the rate constant governed by phosphotransfer and product release.

We have shown that conformational cycling of the C-terminal tail is essential for CDK9 to proceed through an ordered kinetic pathway (Figure 5). We propose that the DRB-bound structure of CDK9/cyclin T reported here represents a short-lived conformational state that is adopted during the phosphorylation cycle. The C terminus transiently folds over the ATP binding site, thereby stabilizing the N- and C-terminal kinase lobes and promoting ATP binding. The relevance of this structure for the catalytic mechanism was corroborated by mutation of contacting residues in the CDK9 tail. Conformational transitions are essential elements of the reaction mechanisms of protein kinases, and the closing together of the N- and C-terminal lobes is a characteristic structural feature of protein kinase activation (Huse and Kuriyan, 2002). Like CDK9, protein kinase A (PKA), the prototypic protein kinase, undergoes a rearrangement of its C-terminal tail to stabilize a closed kinase conformation (Hyeon et al., 2009). Although the C-terminal sequences of CDK9 and PKA are unrelated in sequence and structure, both play a role in stabilizing the kinase conformation during the catalytic cycle.

Studies of the kinetic mechanisms of a number of protein kinases have consistently shown that these kinases go through a ternary complex rather than employing a “ping-pong” mechanism to effect phosphotransfer. Upon further examination, however, the kinetic mechanisms appear to differ as to whether substrate binding and product release follow an ordered or random pathway (Clare et al., 2001; Konstantinidis et al., 1998). PKA has been most extensively studied in order to identify the protein kinase-catalyzed rate-limiting step. This enzyme has been shown to follow a reaction pathway in which the chemical step is fast and the rate-limiting step is the release of ADP (Zhou and Adams, 1997). Our results are compatible with the notion that this step is also rate limiting for P-TEFb turnover.

Of note, although they are closely related in sequence and structure, members of the CDK family do not follow the same kinetic pathway. Studies on CDK2 and CDK5 have demonstrated that ATP and peptide substrate binding is random (Clare et al., 2001), whereas CDK4 proceeds through an ordered sequential mechanism in which ATP is the first substrate to be bound and the phosphoprotein is the last product to be released (Konstantinidis et al., 1998). Our results suggest that CDK9 catalysis also proceeds through an ordered mechanism but differs from CDK4 in that after the catalytic step, the phosphorylated protein is the first product to leave the enzyme active site.

The Pol II CTD presents CDK9 with multiple phosphorylation sites on a single polypeptide. A mechanism that releases the protein product prior to releasing ADP disfavors scanning of the protein substrate and may therefore affect the distribution of phosphorylated Ser2 sites throughout the CTD, especially if the rate of ADP dissociation is slow. Thus, an ordered mechanism would be predicted to lead to phosphorylation of discrete sites distributed along the CTD, rather than to sequential phosphorylation of consecutive sites.

It was recently reported that p-TEFb can phosphorylate Ser5 on the CTD substrate in vitro and is unable to phosphorylate a CTD that has been prephosphorylated at Ser5 or Ser2 (Czudnochowski et al., 2012). Our models of peptide-bound CDK9 support the notion of a Ser2/Ser5 dual specificity and can to some extent rationalize the observation that there are no Ser2 and Ser5 phosphorylations in the same repeat (see Results section of the Supplemental Information). The CDK9 Ser2 specificity observed in vivo may be due to additional factors or the C-terminal sequence in cyclin T that are absent in the in vitro system. Further results reported by Czudnochowski et al. (2012) support a mechanism of CTD phosphorylation by P-TEFb that is distributive. The results presented here are consistent with such a model.

The CTD serves as a binding platform for transcription and RNA processing factors, and the phosphorylation pattern of the multiple repeats determines which factors bind. Recently, it was shown that in yeast, the transcription termination factors Rtt103 and Pcf11 achieve high affinity and specificity both by specifically recognizing the Ser2 phosphorylated CTD and by cooperatively binding to neighboring CTD repeats. This cooperativity is thought to ensure that binding is confined only to authentic polyadenylation sites where Ser2 phosphorylation density is highest (Lunde et al., 2010). CDK9 begins to phosphorylate the CTD close to the 5′ end of the gene, and at this stage it may be advantageous for the cell to phosphorylate only discrete repeats rather than most or all repeats. Recently, CDK12 and CDK13 were identified as CTD Ser2 kinases and metazoan orthologs of yeast Ctk1 (Bartkowiak et al., 2010; Blazek et al., 2011). In contrast to CDK9, which locates to the 5′ end of the gene, CDK12 occupancy increases toward the 3′ end and may account for the high Ser2 phosphorylation in this region.

Sequence alignment shows that CDK7–CDK9, CDK12, and CDK13 all have an extended C-terminal tail beyond the canonical protein kinase fold. A prediction of our model is that although these regions are not conserved in sequence, they could impose a shared kinetic mechanism that would also directly contribute to the pattern of CTD phosphorylation. Upon loss of the C-terminal tail, the CDK9 sequence is now similar to CDK2 and CDK5, both of which encode little more than the core protein kinase catalytic fold. Like these shorter CDKs, CDK9_330_ follows a random substrate-binding model.

In vivo, the CDK9 C-terminal tail is (auto)phosphorylated at several positions (http://www.phosida.com; Gnad et al., 2011). Although our experiments using prephosphorylated CDK9 have shown that the phosphorylation status of the CDK9 tail does not affect P-TEFb activity in vitro (Baumli et al., 2008), phosphorylation of the CDK9 C-terminal tail is required for the binding of HIV TAR RNA to P-TEFb (Garber et al., 2000). It remains to be determined whether this phosphorylation also affects the recruitment of endogenous regulatory proteins. A further prediction from our results is that the binding of such proteins to the CDK9 C-terminal tail could change the kinetic mechanism of P-TEFb. This change could in turn alter the phosphorylation patterns on the Pol II CTD and the complement of transcription factors bound to it.

## Experimental Procedures

### Protein Variants, Expression, and Purification

C-terminally truncated (residues 1–330, CDK9_1-330_) and full-length CDK9 (CDK9_FL_) in complex with cyclin T (residues 1–259, cyclin T_259_; residues 1–288, cyclin T) were expressed and purified as previously described (Baumli et al., 2008). Cyclin T constructs that are optimal for crystallization and contain the mutations Q77R/E96G/F241L were used throughout. We previously characterized this variant crystallographically and enzymatically (Baumli et al., 2012). The validity of the kinetic mechanism described in this work was confirmed using cyclin T encoding for the wild-type sequence. Recombinant baculoviruses for generation of the C-terminally truncated CDK9 proteins were generated using the BD Baculo gold system (BD Biosystems). The CDK9 mutants were prepared using the QuikChange XL kit (Stratagene) according to the manufacturer's instructions. All constructs were verified by DNA sequencing. A GST-tagged construct of the Pol II CTD encoding residues C-terminal of residue 1586 (GST-CTD; further details are provided in the Supplemental Experimental Procedures section of the Supplemental Information) was expressed in BL21 DE3 cells overnight in TB medium at 18°C after induction with 0.2 mM isopropylthio-β-D-thiogalactoside (IPTG). The cell pellets were resuspended in CTD buffer (20 mM Tris pH 8.0, 300 mM NaCl, 5 mM dithiothreitol [DTT], and complete protein inhibitor cocktail [Roche]), lysed by sonication, and purified by glutathione affinity chromatography.

### Crystallization and Structure Determination

CDK9_FL_/cyclin T_259_ was concentrated to 4 mg/ml in a buffer containing 20 mM Tris pH 8.0, 500 mM NaCl, 10% glycerol, and 5 mM DTT, and crystallized at 4°C in sitting drops. CDK9_FL_/cyclin T_259_ was crystallized in 1% PEG 1K, 180–200 mM sodium/potassium phosphate pH 6.2, 4 mM tris(2-carboxyethyl)phosphine) (TCEP). To prepare the DRB complex, preformed crystals were soaked overnight in 3.6% PEG 1K, 162 mM sodium/potassium phosphate pH 6.2, 4 mM TCEP, 23.4% glycerol, and 200 μM DRB (resuspended in water), and then cryocooled in liquid nitrogen. Data were collected for the CDK9_FL_/cyclin T_259_/DRB and CDK9_FL_/cyclin T_259_ complexes at beamlines ID29 and ID14-4 of the European Synchrotron Radiation Facility. Data were processed and integrated using MOSFLM and SCALA to 3.6 and 3.2 Å resolution, respectively. For the CDK9_FL_/cyclin T_259_/DRB crystal structure, strong electron density corresponding to the inhibitor was seen at the CDK9 ATP binding site after initial rigid-body and TLS refinement using PHENIX.refine (Adams et al., 2010) and using the CDK9_330_/cyclin T_259_ apo structure (PDB: 3BLH) as the initial model. Final models were generated by alternative cycles of rebuilding in COOT (Emsley and Cowtan, 2004) and refinement using PHENIX.refine. Invariant regions in the CDK9_FL_/cyclin T_259_/DRB structure were refined by rigid-body and TLS refinement. The glycine-rich loop, the αC-β3 loop, and the C terminus (residues 325–340) of CDK9, as well as cyclin T_259_ residues 150–157, were built in COOT, and coordinate and B-factor refinement was performed on those regions only.

### Kinase Assays

CDK9/cyclin T activity was measured by incorporating radiolabeled phosphate into substrate GST-CTD containing 52 heptad repeats. Seven to seventy five micromolar substrate was incubated with 13 nM CDK9/cyclin T variants. The final reaction conditions contained 150 mM NaCl, 10 mM MgCl_2_, 50 mM HEPES pH 7.5, 2 mM DTT, and γ-^32^P-labeled ATP as indicated. The reaction mixture was incubated for 5 min at 30°C and terminated by the addition of sodium dodecyl sulfate (SDS) sample buffer. Samples were analyzed by SDS-PAGE and subjected to autoradiography. At least three independent measurements were done for each point in any experiment. Results were confirmed in independent experiments. Curves were fitted using the software Graphpad Prism version 5.02 (http://www.graphpad.com) and nonlinear regression as described in the Supplemental Experimental Procedures section of the Supplemental Information.

### Modeling of a CDK9/Cyclin T/Substrate Complex

The polypeptide backbone of the CDK9_FL_/cyclin T_259_/DRB and CDK2/cyclin A/peptide (1QMZ) complexes were superimposed on the C-terminal kinase domain (residues 111–315 and 88–286, respectively) and the substrate peptide was copied into the CDK9/cyclin T_259_ structure. The sequence of the CDK2 substrate peptide was subsequently modified to reflect the sequence of the heptad repeat with Ser2 occupying the phospho-acceptor position.

## Accession Numbers

The atomic coordinates and structure factors have been deposited in the Protein Data Bank (http://www.pdb.org) with accession codes 4EC8 (CDK9FL-cyclin T-DRB) and 4EC9 (CDK9FL-cyclin T).

## Supplemental Information

Document S1. Supplemental Results, Supplemental Experimental Procedures, Figures S1–S4, and Table S1
