# Supplementary material for: The CDK9 Tail Determines the Reaction Pathway of Positive Transcription Elongation Factor b
Source: Structure. 2012 Oct 10;20(10):1788–95. doi: 10.1016/j.str.2012.08.011 (PMC3469819; doi:10.1016/j.str.2012.08.011)
Supplement: Document S1. Supplemental Results, Supplemental Experimental Procedures, Figures S1–S4, and Table S1 [file mmc1.pdf]

Structure, Volume *20*

Supplemental Information

The CDK9 Tail Determines the Reaction Pathway of Positive Transcription Elongation Factor b

Sonja Baumli, Alison J. Hole, Lan-Zhen Wang, Martin E.M. Noble, and Jane A. Endicott

Inventory of Supplemental Information

Supplemental Results: **The CDK9 substrate peptide model supports a dual Ser2/Ser5 specificity**

Supplemental Experimental Procedures: **Describes viruses and plasmids used to produce proteins in this study as well as curve fitting procedures performed to analyze the kinetic data**

Figure S1: **Enzymatic activity of CDK9FL and CDK9330**

Figure S2: **Curves used in the kinetic analysis of CDK9FL**

Figure S3: **Inhibition of CDK9FL and CDK9DA by ADP**

Figure S4: **Conformation of the CDK9 C-terminal tail in different crystal structures**

Table S1: **Apparent kinetic parameters of CDK9 variants**

Supplemental References

Supplementary Results:

The CDK9 substrate peptide model supports a dual Ser2/Ser5 specificity

Recent reports suggest that p-TEFb can phosphorylate Ser5 on the CTD substrate *in vitro* and is unable to phosphorylate a CTD that has been pre-phosphorylated at Ser5 or Ser2 (Czudnochowski et al., 2012). To investigate the structural basis of the observed CDK9 peptide recognition observed we generated a CDK9 substrate peptide model. Based on the peptide-substrate-bound structure of CDK2 (PDB code 1qmz), we have modelled either Ser2 or Ser5 of the CTD heptad repeat into the CDK9 phospho-acceptor site. The CDK2 template contains a hepta-peptide substrate with sequence $H_2H_3A_4S_5P_6R_7K_8$. When Ser2 of a CTD heptad is modelled into the phospho-acceptor site, a putatively phosphorylated Ser5 is modelled to sit in the position equivalent to K_8, immediately adjacent to the phosphorylated serine residue in the CDK9 activation segment. We would expect this to cause an unfavourable juxtaposition of two negatively charged groups, explaining how phosphorylation of Ser5 might inhibit phosphorylation of Ser2. Conversely, when Ser5 of a CTD heptad is modelled into the phospho-acceptor site, a putatively phosphorylated Ser2 is modelled to sit in the position equivalent to H_2, in a largely solvent exposed site. As such, the structure does not immediately suggest how phosphorylation of Ser2 might disfavour phosphorylation of Ser5.

Supplementary Methods:

Viruses and Plasmids: Viruses and plasmids used for the expression of CDK9/cyclin T are as described (Baumli et al., 2008). The plasmid used to produce GST-CTD encoded the following protein sequence (rpb1 residues 1586-1970 in bold):

RGS**GGAMSPSYSPTSPAYEPRSPGGYTPQSPSYSPTSPSYSPTSPSYSPTSPNYSPTSPSYSPTSPSYSPT**
SPSYSPTSPSYSPTSPSYSPTSPSYSPTSPSYSPTSPSYSPTSPSYSPTSPSYSPTSPSYSPTSPSYSPTSPSYS
PTSPSYSPTSPSYSPTSPNYSPTSPNYTPTSPSYSPTSPSYSPTSPNYTPTSPNYSPTSPSYSPTSPSYSPTS
PSYSPSSPRYTPQSPTYTPSSPSYSPSSPSYSPTSPKYTPTSPSYSPSSPEYTPASPKYSPTSPKYSPTSPKY
SPTSPTYSPTTPKYSPTSPTYSPTSPVYTPTSPKYSPTSPTYSPTSPKYSPTSPTYSPTSPKGSTYSPTSPGY
SPTSPTYSLTSPAISPDDSDEENEFPGRLERPH

Curve fitting: Kinetic constants were calculated by fitting of the data using non linear regression in GraphPad Prism version 5.02 (www.graphpad.com) and the predefined equations corresponding to competitive, non competitive, uncompetitive and mixed inhibition. The equations used to fit the data corresponding to the ordered ternary complex mechanism or the substituted enzyme mechanisms were defined as:

$$v = \frac{V_{max}\,[ATP][GST\text{-}CTD]}{K_{ATP}K_{M,\,GST\text{-}CTD} + K_{M,GST\text{-}CTD}[ATP] + K_{M,\,ATP}[GST\text{-}CTD] + [ATP][GST\text{-}CTD]}$$

for the ordered ternary complex mechanism, and

$$v = \frac{V_{max}\,[ATP][GST\text{-}CTD]}{K_{M,GST\text{-}CTD}[ATP] + K_{M,\,ATP}[GST\text{-}CTD] + [ATP][GST\text{-}CTD]}$$

for the substituted enzyme mechanism. [ATP] and [GST-CTD] represent the corresponding concentrations, and K_{ATP}, $K_{M,\,GST\text{-}CTD}$ and $K_{M,\,ATP}$ correspond to the ATP dissociation constant, and the Michaelis constants for GST-CTD and ATP respectively.

Supplementary Figures

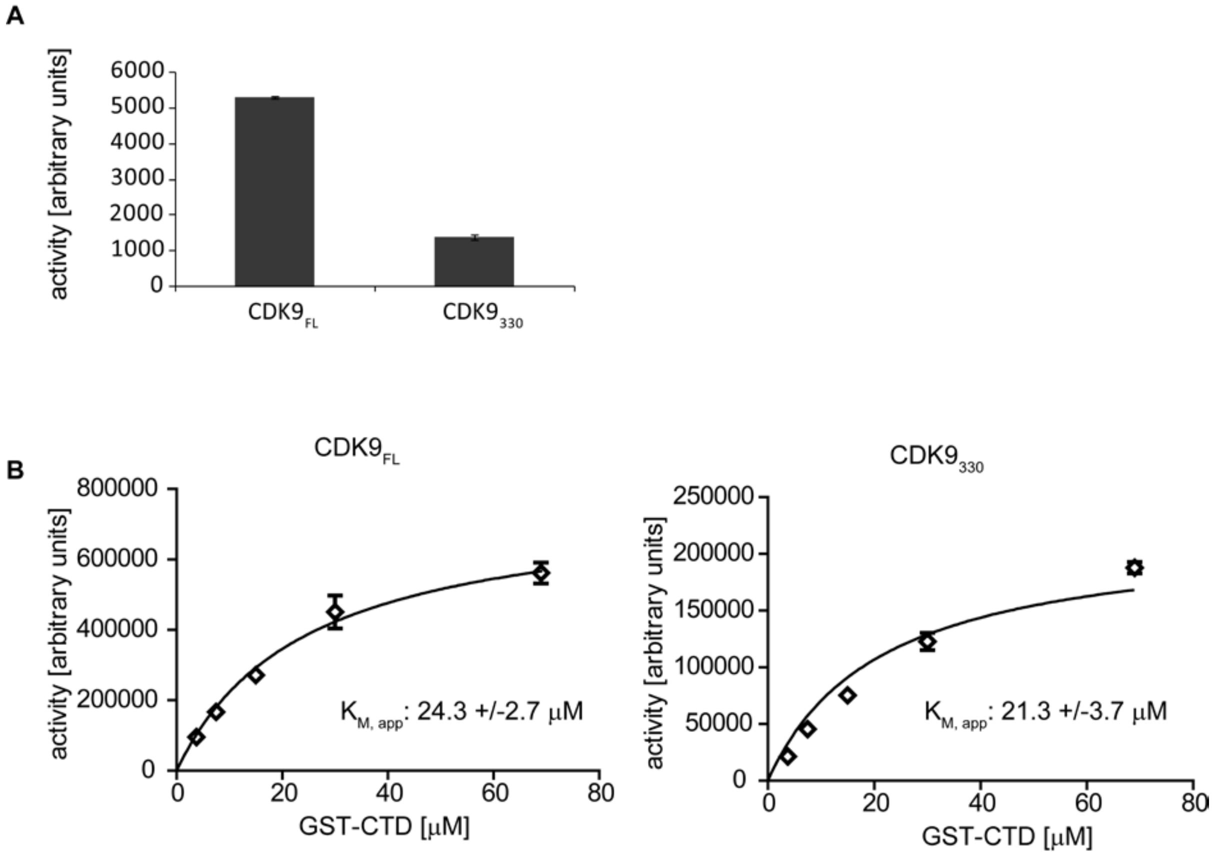

Supplementary Figure 1, associated with Figure 1. Enzymatic activity of $CDK9_{FL}$ and $CDK9_{330}$. **(a)** Activity of $CDK9_{FL}$ and $CDK9_{330}$ towards GST-CTD (37 μM) at 100 μM ATP. **(b)** K_M determination for $CDK9_{FL}$ and $CDK9_{330}$ at 100 μM ATP concentration. All measurements were done in triplicates. Error bars indicate standard errors. The result was confirmed in several independent experiments.

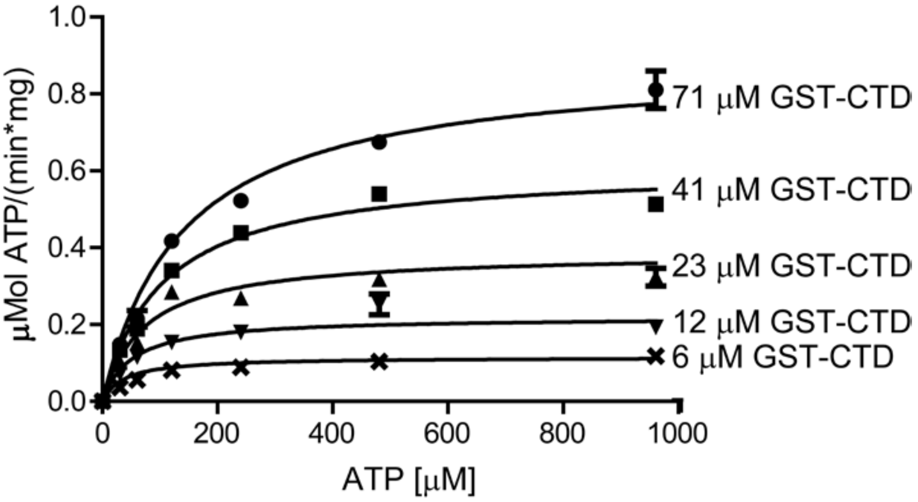

Supplementary Figure 2, associated with Table I. Kinetic analysis of $CDK9_{FL}$. Michaelis-Menten plot of velocity *versus* ATP concentration in the assay is shown for different GST-CTD substrate concentrations indicated on the right. All measurements were done in triplicates. Average values and standard errors are shown. The result was confirmed in independent experiments. The curves represent the combined data fitted to a steady state ordered recruitment ternary complex model and correspond to the values given in Table I.

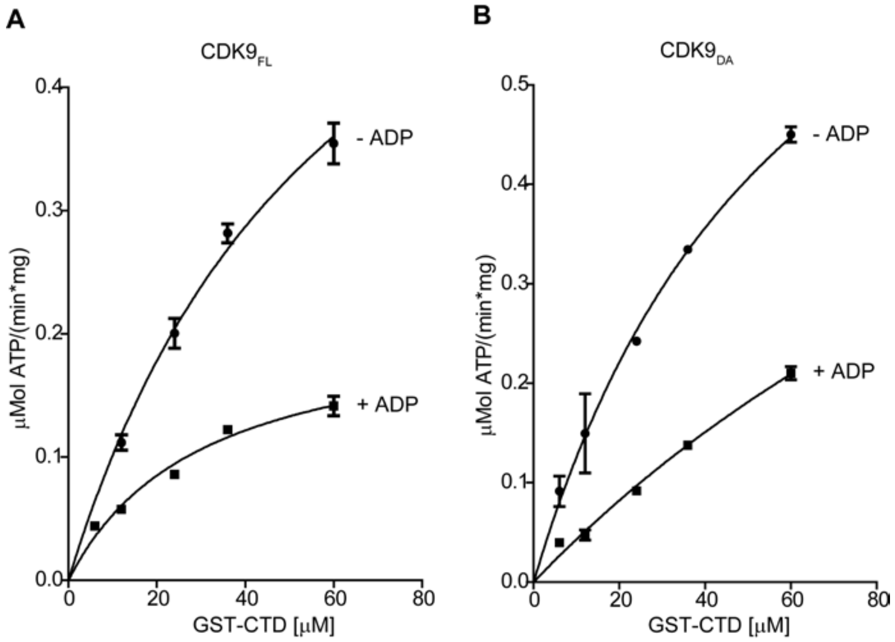

Supplementary Figure 3, associated with Figure 2. Inhibition of $CDK9_{FL}$ and $CDK9_{DA}$ by ADP. **(a)** Activity of $CDK9_{FL}$/cyclin T towards increasing amounts of the CTD at an ATP concentration of 100 μM in the absence or presence of 5 μM ADP. Curves were fitted to either a competitive inhibition or non competitive inhibition model and compared by Akaike's information criterion. Curves shown are fitted to a non-competitive inhibition model (probability: 99.8%). **(b)** Activity of $CDK9_{DA}$/cyclin T towards increasing amounts of the CTD is shown as in (a). Curves shown are fitted to a competitive inhibition model (probability: 98.0%). All measurements were done in triplicates and the result confirmed in independent experiments. Error bars represent standard errors.

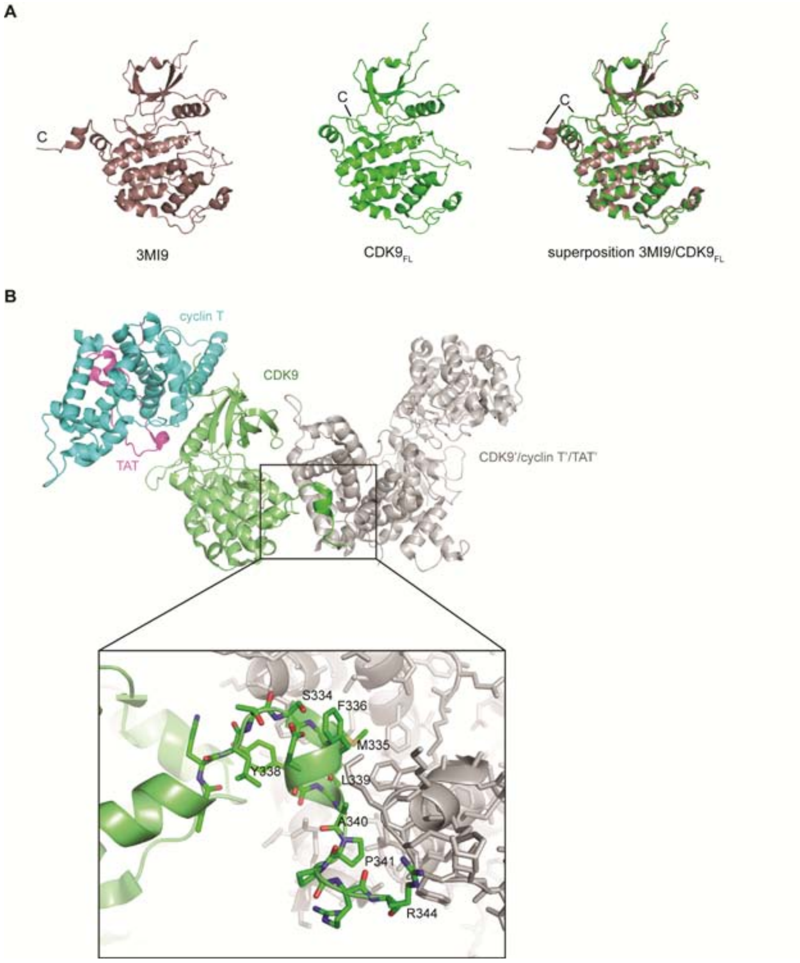

Supplementary Figure 4, associated with Figure 3: Conformation of the CDK9 C-terminal tail in different crystal structures. **(a)** CDK9 structure as observed in the CDK9/cyclin T/TAT complex (PDB: 3MI9, brown) and the CDK9FL/cyclin T/DRB structures (this study, green). The superposition illustrates the different conformations adopted by the CDK9 tail in the two CDK9 complex structures. The three panels present the same view for each CDK9 structure. **(b)** The conformation of the CDK9 C-terminal tail in the CDK9/cyclin T/TAT structure is determined by crystal contacts to a neighbouring molecule. One P-TEFb unit consisting of CDK9 (green) cyclin T (blue) and TAT (pink) is shown in colour, the symmetry related complex in the crystal is shown in grey. An enlargement of the interface illustrates the details of the interaction. CDK9 residues within 4Å of a symmetry related molecule are labelled. Contacts between the central and symmetry related amino acid residues (indicated here with a prime) include: CDK9 S334 to cyclinT W207’; CDK9 M335 to cyclinT W207’, cyclinT F176’ and TAT F32’; CDK9 F336 to TAT K28’; CDK9 Y338 to cyclinT R51’ and cyclinT W207’; CDK9 L339 to cyclinT R251’ and cyclinT L245’; CDK9 A340 to TAT K28’ and TAT F32’; CDK9 P341 to TAT A42’; CDK9 R344 to cyclinT K261’ and TAT C26’.

Supplementary Tables

	$K_{M,app\ (ATP)}$ [μM]	$V_{max,app\ (ATP)}$ [μMol(min*mg)$^{-1}$]	$K_{cat,app\ (ATP)}$ [min^{-1}]	$K_{cat,app\ (ATP)}$ /$K_{M,app\ (ATP)}$ [μM^{-1}min^{-1}]
$CDK9_{FL}$	160 +/- 13	0.73 +/- 0.02	56 +/- 1.5	0.35 +/- 0.03
$CDK9_{330}$	1853 +/- 278	4.9 +/- 0.05	350 +/- 3.6	0.19 +/- 0.03

Kinase variant	$V_{max,app\ (CTD)}$ [μMol(min*mg)$^{-1}$]	$K_{cat,app\ (CTD)}$ [min^{-1}]	$K_{cat,app\ (CTD)}$/$K_{m,app\ (CTD)}$ [μM^{-1}min^{-1}]
$CDK9_{FL}$	0.53 +/- 0.02	40.8 +/- 1.3	1.7 +/- 0.05
$CDK9_{350}$	0.36 +/- 0.01	26.5 +/- 0.7	1.1 +/- 0.05
$CDK9_{340}$	0.25 +/- 0.01	18.1 +/- 0.7	0.75 +/- 0.04
$CDK9_{330}$	0.17 +/- 0.004	12.1 +/- 0.3	0.5 +/- 0.01

Supplementary Table I, associated with Figure 1

Upper panel: apparent kinetic parameters towards ATP substrate for the different CDK9/Cyclin T variants. Experiments were done at a concentration of 66 μM GST-CTD. Errors are given as standard errors. Lower panel: apparent kinetic parameters for the different CDK9/Cyclin T variants towards the substrate GST-CTD. Experiments were done at a concentration of 100 μM ATP and data were fitted assuming a common $K_{M\ (GST\text{-}CTD)}$ of 24.3 μM for all kinase variants. All values correspond to the curves shown in Fig. 1b. Errors represent standard errors.

Supplementary References

Baumli, S., Lolli, G., Lowe, E.D., Troiani, S., Rusconi, L., Bullock, A.N., Debreczeni, J.E., Knapp, S., and Johnson, L.N. (2008). The structure of P-TEFb (CDK9/cyclin T1), its complex with flavopiridol and regulation by phosphorylation. Embo J *27*, 1907-1918.

Czudnochowski, N., Bosken, C.A., and Geyer, M. (2012). Serine-7 but not serine-5 phosphorylation primes RNA polymerase II CTD for P-TEFb recognition. Nat Commun 3, 842.
